# A mitoxyperilysis-related signature stratifies prognosis and identifies an aggressive colorectal cancer ecosystem with immune remodeling

**DOI:** 10.3389/fcell.2026.1851988

**Published:** 2026-07-03

**Authors:** Yi Zhang, Bin Sun, Joshua Lin, Chengsheng Ding, Xueliang Zhou, Ximo Xu, Kefan Dai, Xiaodong Fan, Man Lu, Zirui He, Xiao Yang, Minhua Zheng

**Affiliations:** 1 Department of General Surgery, Ruijin Hospital, Shanghai Jiao Tong University School of Medicine, Shanghai, China; 2 Department of General Surgery, Shanghai General Hospital, Shanghai Jiao Tong University School of Medicine, Shanghai, China; 3 First Clinical Medical College, Nanjing University of Chinese Medicine, Nanjing, China; 4 Shanghai Institute of Digestive Surgery, Ruijin Hospital, Shanghai Jiao Tong University School of Medicine, Shanghai, China; 5 Department of General Surgery, Loujiang New City Hospital of Taicang (Taicang Branch of Ruijin Hospital Affiliated with Shanghai Jiao Tong University School of Medicine), Suzhou, China

**Keywords:** artificial intelligence, cancer ecosystem state, cell-cell communication, colorectal cancer, machine learning, mitoxyperilysis, single-cell RNA sequencing, tumor microenvironment

## Abstract

**Background:**

Mitoxyperilysis is a recently described lytic cell death pathway linked to innate immune activation and metabolic disruption. Its clinical and biological relevance in colorectal cancer (CRC) remains unclear. This study aimed to develop a mitoxyperilysis-related signature (MRS) for prognostic stratification and to characterize its associated tumor microenvironmental features.

**Methods:**

Bulk transcriptomic and clinical data from TCGA, GSE17536, and GSE29621 were used to construct and validate the MRS through an integrated machine-learning framework. Survival analysis, nomogram construction, pathway enrichment, immune deconvolution, ESTIMATE analysis, immunophenoscore-related assessment, and drug-response prediction were performed. Single-cell RNA sequencing and cell-cell communication analyses were used to define the cellular context of the MRS-associated phenotype. ANO1 was further evaluated by pan-cancer and CRC-specific analyses and validated by *in vitro* and *in vivo* experiments.

**Results:**

The MRS consistently stratified overall survival across the training and validation cohorts and improved individualized risk prediction when incorporated into a nomogram. The MRS-high subtype was associated with extracellular matrix remodeling, invasion-related pathways, stromal enrichment, immune infiltration, and increased expression of inhibitory immune checkpoints. Single-cell analysis linked the adverse phenotype to epithelial cells with higher proliferative, metastatic, stress-adaptive, and immune-interactive features. These cells showed enhanced communication with stromal and immune compartments, particularly through FN1-, MK-, CXCL-, and CCL-related signaling. ANO1 was identified as a candidate downstream effector associated with the MRS-high state. Functionally, ANO1 knockdown suppressed CRC cell proliferation, migration, invasion, and xenograft tumor growth.

**Conclusion:**

Mitoxyperilysis-related transcriptional programs are associated with a clinically aggressive and immune-remodeled ecosystem state in colorectal cancer. The MRS serves as a reliable tool for prognostic stratification and provides a transcriptional framework for characterizing CRC states linked to epithelial aggressiveness, microenvironmental remodeling, intercellular communication, and adverse clinical outcome. ANO1 may function as an important downstream effector associated with this MRS-high ecosystem state.

## Introduction

Colorectal cancer (CRC) continues to be one of the most prevalent malignant neoplasms worldwide and represents a major contributor to cancer-related deaths on a global scale (Sung et al., 2021). Although considerable efforts have been devoted to population screening, surgical interventions, systemic therapies, and biomarker-guided clinical management, the survival outcome for patients with late-stage CRC remains unsatisfactory (Cervantes et al., 2023). This poor prognosis is largely attributable to the biological heterogeneity of CRC and the highly dynamic nature of its tumor microenvironment (TME) (Hanahan, 2022). It is evident that malignant epithelial cells within CRC interact extensively with fibroblasts, endothelial cells, myeloid cells, and lymphoid populations in a structurally and functionally complex manner, giving rise to an intricate tumor ecosystem (Pelka et al., 2021). This ecosystem is further shaped by extracellular matrix (ECM) remodeling, inflammatory signaling, and metabolic stress (de Visser and Joyce, 2023), which collectively drive local invasion, metastatic dissemination, and therapeutic resistance (An et al., 2024). Notably, these factors also contribute to substantial variability in the efficacy of immunotherapy in CRC (Yu et al., 2024). While immune checkpoint blockade has reshaped the treatment paradigm for mismatch repair-deficient or microsatellite instability-high CRC, most CRCs still derive little benefit from currently available immunotherapeutic options (André et al., 2020; Han et al., 2024; Yamamoto et al., 2024). This unmet need has fueled increasing attention to the biological processes that couple tumor-intrinsic stress programs with immune and stromal remodeling (Hamid et al., 2024; Liu J. et al., 2024).

Accumulating evidence suggests that metabolic stress is a key determinant of tumor progression and immune contexture in CRC (Chen et al., 2024). Mitochondrial dysfunction, nutrient deprivation, redox imbalance, and altered lipid metabolism are no longer viewed as passive consequences of tumor growth (Meyiah et al., 2025). Rather, these states actively reshape both malignant cell behavior and the non-malignant compartments of the TME (Liu W. et al., 2024). In parallel with these stress-related processes, regulated cell death (RCD) has gained increasing attention as an important conceptual framework for understanding how stress adaptation, inflammation, and cancer progression are interconnected (Saxena et al., 2024). Moreover, different RCD programs are closely associated with prognosis, therapeutic vulnerability, and the immunological landscape of tumors (De Silva et al., 2024). This is particularly evident in CRC, where oxidative stress, chronic inflammation, stromal activation, and immune dysfunction coexist across different stages of disease progression (Andac-Aktas and Calibasi-Kocal, 2025).

Recently, Wang et al. identified mitoxyperilysis as a distinct lytic cell death pathway induced by the synergism of innate immune activation and metabolic disruption (Wang et al., 2025). Unlike canonical pyroptosis, apoptosis, necroptosis, or ferroptosis, mitoxyperilysis is driven by BAX/BAK1/BID-dependent mitochondrial damage, oxidative stress, glutathione depletion, prolonged mitochondria-plasma membrane contact, local membrane lipid peroxidation, and eventual membrane rupture (Wang et al., 2025). The related concept of mitoxyperiosis further emphasizes the critical role of prolonged mitochondria-membrane contact in this process (Casadio, 2026). This mechanism is especially intriguing in CRC, because mitochondrial stress, inflammatory signaling, and metabolic instability are all prominent features of the CRC microenvironment (Hu S. et al., 2025). However, the clinical and biological significance of mitoxyperilysis-associated programs in CRC remains unknown (Kaviyarasan et al., 2024).

It is not yet clear whether mitoxyperilysis-related transcriptional patterns can stratify prognosis in CRC (Li et al., 2025). It is also unknown whether such patterns define biologically meaningful tumor states linked to ECM remodeling and immune dysfunction (Wang et al., 2024). Moreover, the cellular basis of these programs and their downstream effectors remain to be clarified (George and Johdi, 2025). In the present study, we established a mitoxyperilysis-related risk signature (MRS) for CRC using an integrated machine-learning framework. We then investigated its association with prognosis, matrix remodeling, immune-stromal reprogramming, epithelial-state heterogeneity, and intercellular communication. Finally, we identified ANO1 as a candidate downstream effector associated with the MRS-high phenotype and validated its functional role in CRC progression. Collectively, this study extends the concept of mitoxyperilysis from a newly described cell death mechanism to a clinically informative systems-level framework for understanding aggressive and immune-remodeled CRC.

## Materials and methods

### Study design and data collection

**FIGURE 1:**
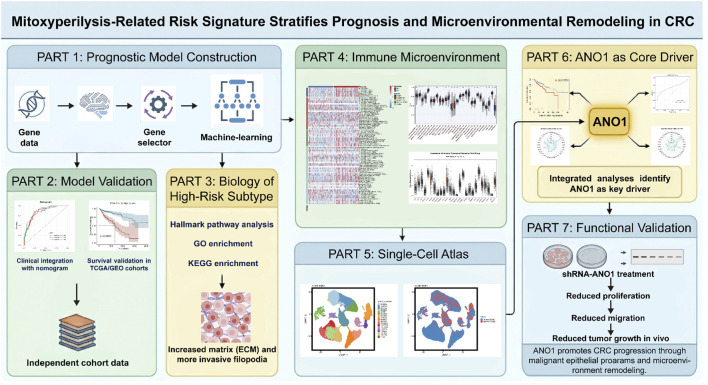
The flowchart of the research.

This work aimed to delineate molecular characteristics linked to an aggressive, immune-remodeled form of colorectal cancer (CRC). To address this objective, we implemented a multilevel analytic scheme that integrated bulk-transcriptome interrogation, machine-assisted prognostic modeling, appraisal of the tumor microenvironment, single-cell transcriptomic dissection, inference of intercellular signaling, pan-cancer scrutiny of ANO1, and downstream verification in cultured cells as well as murine models. A schematic synopsis of the entire workflow is presented in Figure 1. Bulk RNA-expression matrices and corresponding survival annotations for colorectal cancer were assembled from the TCGA discovery cohort and two external datasets, GSE17536 and GSE29621, to enable independent corroboration. In parallel, single-cell transcriptional profiles derived from CRC specimens and their matched non-neoplastic tissues were leveraged to pinpoint the cellular substrate underlying the unfavorable risk phenotype. In parallel, pan-cancer datasets were analyzed to assess ANO1 expression, prognostic relevance, immunogenomic correlations, and microenvironment-associated features across tumor types.

### Bulk transcriptomic preprocessing

Bulk RNA expression matrices and matched clinical metadata were retrieved from the UCSC Xena and GEO repositories. Cases without complete survival records or key clinicopathological information were removed before analysis. Expression values were transformed using log2 (TPM+1), and gene annotations were standardized to official gene symbols for subsequent analyses. In GEO cohorts, when several probes corresponded to a single gene, their values were averaged to generate one expression value per gene. Where necessary, batch variation between platforms was corrected using the ComBat algorithm.

### Construction of the prognostic signature

**FIGURE 2:**
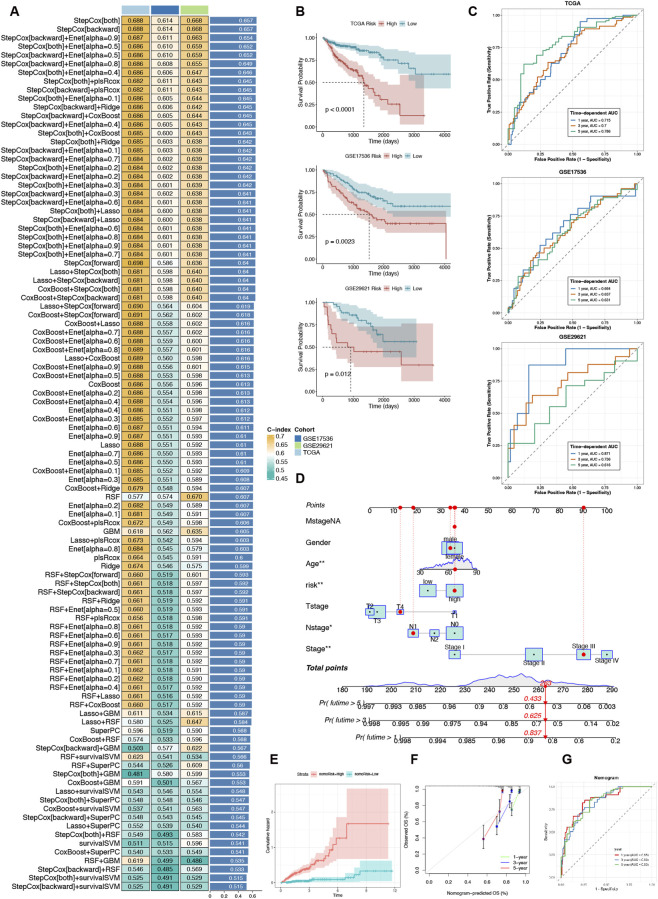
Construction, external validation, and clinical translation of the prognostic signature in colorectal cancer. **(A)** Heatmap summarizing the performance of candidate machine-learning algorithm combinations across the TCGA, GSE17536, and GSE29621 cohorts, evaluated using Harrell’s concordance index (C-index). The optimal model with the most robust cross-cohort performance was selected for subsequent analyses. **(B)** Kaplan-Meier survival curves showing overall survival differences between the high-risk and low-risk groups in the TCGA training cohort and the two external validation cohorts (GSE17536 and GSE29621). **(C)** Time-dependent receiver operating characteristic (ROC) curves for predicting 1-, 3-, and 5-year overall survival in the TCGA, GSE17536, and GSE29621 cohorts. **(D)** Nomogram integrating the risk score with clinicopathological variables for individualized prediction of 1-, 3-, and 5-year overall survival. **(E)** Cumulative hazard curves stratified by nomogram-defined risk groups. **(F)** Calibration plots showing the agreement between nomogram-predicted and observed overall survival probabilities at 1, 3, and 5 years. **(G)** Time-dependent ROC curves evaluating the discriminative performance of the nomogram.

For construction of the CRC prognostic classifier, putative genes were funneled into an integrated machine-learning pipeline that accommodated a spectrum of modeling strategies. As outlined in Figure 2A, the algorithmic search domain comprised StepCox, Enet, Lasso, Ridge, CoxBoost, RSF, GBM, SuperPC, the plsRcox method, and survival-SVM, with models assessed either as stand-alone approaches or in hybridized combinations. Their predictive behavior was then benchmarked in the TCGA discovery set together with the independent validation datasets by means of Harrell’s C-index. The configuration that displayed the steadiest cross-cohort performance was preserved as the definitive prognostic signature. On that basis, an individualized score was computed from the retained model coefficients, after which cases were dichotomized into lower- and higher-risk subsets using the median value as the cutoff.

### Survival analysis and nomogram construction

Overall survival was compared between risk groups using Kaplan-Meier analysis and the log-rank test. Time-dependent receiver operating characteristic (ROC) curves were generated to assess predictive performance at 1, 3, and 5 years. To improve clinical applicability, the risk score was integrated with conventional clinicopathological variables, including age, sex, T stage, N stage, M stage, and other available covariates, to construct a nomogram for individualized survival prediction. Nomogram performance was evaluated by calibration analysis, cumulative hazard analysis, and time-dependent ROC analysis.

### Functional annotation of the high-risk phenotype

To delineate the molecular programs underlying the unfavorable-risk state, transcriptomic differences between the high- and low-risk strata were assessed with the limma framework, and features with an adjusted P value below 0.05 were regarded as statistically significant. Activity of curated Hallmark pathways was inferred by GSVA with the Hallmark collections downloaded from MSigDB. The resulting differentially expressed genes were subsequently submitted to GO and KEGG over-representation analyses to uncover biological processes and signaling routes preferentially linked to the high-risk subtype. In addition, survival patterns were examined within major clinicopathological subdivisions, including age as well as T, N, and M stage categories.

### Immune infiltration and microenvironmental profiling

To depict the microenvironmental landscape associated with the risk signature, the relative abundance of immune and stromal populations was inferred through several deconvolution tools, namely, TIMER, CIBERSORT, quanTIseq, MCP-counter, and xCell. We then contrasted immune-cell infiltration metrics and immune-signature scores across the two risk strata. Expression levels of representative chemokines together with immune checkpoint-associated genes were further evaluated against the risk score to probe chemokine-linked stromal-immune remodeling and broader immunoregulatory attributes. StromalScore, ImmuneScore, and ESTIMATEScore were derived using the ESTIMATE algorithm. To appraise possible relevance to immunotherapy, IPS-related indicators under distinct immune checkpoint blockade scenarios were compared between groups. A drug-response prediction procedure was also conducted to infer subgroup-specific susceptibility to candidate targeted compounds, with sensitivity estimates generated from GDSC-trained models implemented in the oncoPredict package.

### Single-cell transcriptomic processing and cellular annotation

Single-cell RNA-sequencing profiles derived from CRC specimens and matched normal tissues were analyzed with the Seurat pipeline (v4.4.1). Low-quality cells were discarded using the following filtering rules: nFeature_RNA <500 or >5,000, nCount_RNA <500 or >50,000, or a mitochondrial transcript proportion exceeding 20%. After normalization, highly variable genes were identified, followed by dimensionality reduction through principal component analysis and visualization in UMAP space. Cellular clusters were then resolved with a graph-based unsupervised strategy. Major cell populations were assigned according to canonical markers, including EPCAM/KRT18/KRT19 for epithelial cells, PECAM1/VWF/ENG for endothelial cells, DCN/LUM/COL1A1 for fibroblasts, CD79A/CD79B for B cells, CD3D/CD3E/CD3G for T cells, KLRB1/GNLY/NKG7 for NK cells, and CD68/CD14/LYZ for myeloid cells. In total, 48,891 cells satisfied the quality-control criteria and were retained for subsequent analyses.

### Definition of epithelial high-state and low-state programs

Given that bulk transcriptomic analyses suggested an aggressive and invasion-associated phenotype, we focused on the epithelial compartment for state-level characterization. To ensure consistency between the bulk-derived prognostic stratification and the single-cell state annotation, single-cell transcriptomic profiles were first aggregated into sample-level pseudobulk expression matrices. These sample-level pseudobulk expression matrices were subsequently evaluated with the previously developed mitoxyperilysis-related risk signature (MRS), and an MRS value was assigned to each specimen according to the fixed coefficients of the model. Using the median MRS value as the threshold, samples were divided into MRS-high and MRS-low categories. The resulting sample-level classifications were then traced back to individual cells through their sample-of-origin annotations, such that epithelial cells originating from MRS-high samples were designated as high-state cells, whereas those derived from MRS-low samples were labeled as low-state cells. Stemness, proliferation, and metastasis scores were next computed for each epithelial cell with AddModuleScore and compared across the two states. Genes differentially expressed between high-state and low-state epithelial cells were further analyzed by GO and KEGG enrichment to delineate the biological programs underlying the adverse epithelial phenotype.

### Cell-cell communication analysis

To assess whether the high-state epithelial program was associated with changes in intercellular signaling, ligand-receptor-based communication analysis was conducted using CellChat v2 or a comparable framework. Major annotated cell populations were designated as sender and receiver compartments. For each state, we quantified the total number of predicted interactions, overall interaction strength, outgoing and incoming signaling patterns, and pathway-specific information flow.

### Cross-neoplasm interrogation of ANO1 with a CRC-focused readout

For ANO1-related analyses, the scope of each dataset was defined explicitly. Pan-cancer analyses were performed across TCGA tumor types. TCGA-based CRC-focused public analyses were conducted primarily in the COAD cohort where matched tumor-normal comparison and clinicopathological annotations were available. Bulk transcriptomic validation of the MRS was based on TCGA together with the external CRC cohorts GSE17536 and GSE29621. Single-cell analyses were performed using CRC single-cell datasets GSE132465 and GSE200097. Institutional IHC validation, *in vitro* assays, and xenograft experiments were conducted in the CRC context. ANO1 was prioritized for subsequent scrutiny owing to its conspicuous placement within the integrative analytic schema. To profile its expression landscape, tumor specimens and corresponding non-neoplastic tissues from diverse malignancy types were juxtaposed for differential-expression assessment. Within the COAD cohort, Kaplan-Meier curves were generated to appraise outcome divergence between cases exhibiting elevated ANO1 abundance and those with comparatively attenuated expression. Receiver operating characteristic analysis was further undertaken to gauge how effectively ANO1 distinguished COAD tissue from normal colorectal counterparts. In parallel, gene set enrichment analysis was employed to delineate signaling circuits and biological programs linked to ANO1 expression status. On a pan-cancer scale, Spearman rank-based testing was used to explore the associations of ANO1 with tumor mutational burden, microsatellite instability, StromalScore, ImmuneScore, ESTIMATEScore, and selected immune checkpoint molecules.

### Cell propagation and establishment of stable ANO1-deficient lines

HCT116 and RKO cells obtained from ATCC (Manassas, VA, USA) were propagated at 37 °C under humidified conditions with 5% CO_2_. Depending on the cell line, cultures were maintained in DMEM or RPMI-1640 supplemented with 10% fetal bovine serum together with 1% penicillin-streptomycin. These two CRC cell lines were adopted as experimental platforms for the ensuing functional assays. Lentiviral constructs carrying two distinct shRNAs directed against ANO1 (ANO1-sh1 and ANO1-sh2), along with a non-targeting negative-control shRNA, were procured from Shanghai GeneChem Co., Ltd. Stable ANO1-suppressed cell populations were then generated through lentiviral infection followed by antibiotic-based selection.

### Cell proliferation and colony formation assays

Cell growth was evaluated with a CCK-8 assay. Briefly, 1 × 10^3^ cells were seeded into each well of 96-well plates. Each group contained six technical replicates, and the experiment was repeated independently three times. CCK-8 reagent (Meilunbio) was added at pre-set intervals up to 96 h, and absorbance was measured at 450 nm. For colony formation, 400 cells were seeded into 6-well plates and maintained in complete medium with 10% FBS for 14 days. Colonies were fixed, stained with 0.1% crystal violet for 30 min, and counted.

### Wound-healing and transwell assays

Cell motility was assessed with a wound-closure experiment. In brief, cells were plated into 6-well dishes and grown until they approached full confluence, after which a straight gap was drawn across the monolayer using a 10-μL pipette tip. Floating or loosened cells were cleared by rinsing with PBS, and the remaining cells were subsequently cultured in serum-deprived medium. Microscopic images were acquired at designated intervals, and closure efficiency was calculated according to the formula (A_0_ - A_n_)/A_0_ × 100%. For the Transwell experiments, cells were loaded into the upper inserts in serum-limited medium, using uncoated chambers for migration assessment and Matrigel-pretreated chambers for invasion assessment. Meanwhile, medium containing 20% FBS was placed in the lower compartment to serve as a chemotactic stimulus. After 48 h, cells traversing the membrane were fixed, stained with 0.5% crystal violet, imaged, and counted microscopically. All assays were performed with three biological replicates.

### Xenograft model

*In vivo* tumor-forming capacity was examined in four-week-old male BALB/c nude mice maintained under specific pathogen-free housing conditions. HCT116 cells carrying stable knockdown of ANO1 via shRNA, together with matched control cells expressing NC shRNA, were inoculated into the right inguinal area at a dose of 5 × 10^6^ cells per animal. Mice were assigned to either the control or ANO1-silenced group, and tumor size was measured at predefined time points. Tumor burden was estimated with the equation (length × width^2^)/2, while body mass was tracked during the experimental period. Upon study termination, the animals were euthanized, and the xenograft masses were removed, photographed, and weighed. Details regarding ethical oversight are provided in the Ethics Statement.

### Statistical procedures

All analyses were conducted using R software (v4.4.1) and GraphPad Prism (v10.1.0). Relationships between categorical clinicopathological characteristics and protein-expression status were tested with Fisher’s exact test. Survival relevance was examined through Kaplan-Meier estimation in conjunction with Cox proportional hazards modeling. Continuous data are presented as mean ± SD, based on no fewer than three independent experiments. For comparisons between two conditions, an unpaired Student’s t-test was applied; when more than two groups were involved, differences were evaluated by one-way ANOVA followed by Tukey’s multiple-comparison test. Methods used for omics-oriented analyses or nonparametric testing are indicated in the corresponding figure legends. All significance tests were two-tailed, and a P value below 0.05 was taken to denote statistical significance.

## Results

### Establishment and verification of a mitoxyperilysis-associated prognostic signature in colorectal cancer

We devised an integrative analytic framework to explore both the prognostic impact and the biological roles of mitoxyperilysis in CRC. Starting from mitoxyperilysis-associated genes, we first performed GSVA to score pathway activity in each TCGA sample and retained genes with a Spearman correlation >0.2 with the GSVA score. Next, we conducted differential expression analysis between CRC tumor tissues and adjacent normal tissues, selecting genes with |log_2_ fold change| > 1 and adjusted P < 0.05. Subsequently, we applied univariable Cox regression to these differentially expressed genes and kept those with P < 0.05 as candidate predictors of overall survival. This sequential filtering yielded 35 genes that entered the subsequent machine-learning step. Employing a large-scale framework comprising 101 algorithmic combinations, we evaluated and selected the model with the most consistent cross-cohort performance, as measured by Harrell’s C-index. The optimal model was StepCox [both] (stepwise Cox regression with bidirectional selection), which was used to define the final mitoxyperilysis-related risk signature (MRS) (Figure 2A).

Based on this signature, patients were divided into MRS-high and MRS-low categories. Across the TCGA, GSE17536, and GSE29621 cohorts, individuals in the MRS-high group consistently exhibited significantly poorer overall survival than those in the MRS-low group (Figure 2B). We further assessed the stability of the model by time-varying ROC evaluation. Within the TCGA dataset, the AUC values for overall survival at 1, 3, and 5 years were 0.715, 0.750, and 0.760, respectively. Comparable performance was observed with values of 0.664, 0.651, and 0.637 in the GSE17536 cohort and values of 0.871, 0.736, and 0.616 in GSE29621 (Figure 2C). To enhance clinical applicability, we used the MRS in combination with conventional clinicopathological parameters to construct a nomogram for patient-specific survival estimation (Figure 2D). Cases classified as high risk by this nomogram exhibited a clearly higher cumulative hazard burden (Figure 2E). Calibration plots demonstrated a strong agreement between projected survival probabilities and the observed outcomes (Figure 2F). The nomogram achieved a C-index of 0.821 (95% CI, 0.779–0.862) and maintained solid discriminative performance in time-dependent ROC assessment (Figure 2G). Collectively, these findings provide concrete evidence of robustness of the MRS across independent cohorts and highlight its potential as an interpretable tool for prognostic assessment in clinical settings.

### The high-risk subtype is characterized by extracellular matrix remodeling and invasion-associated biological programs

**FIGURE 3:**
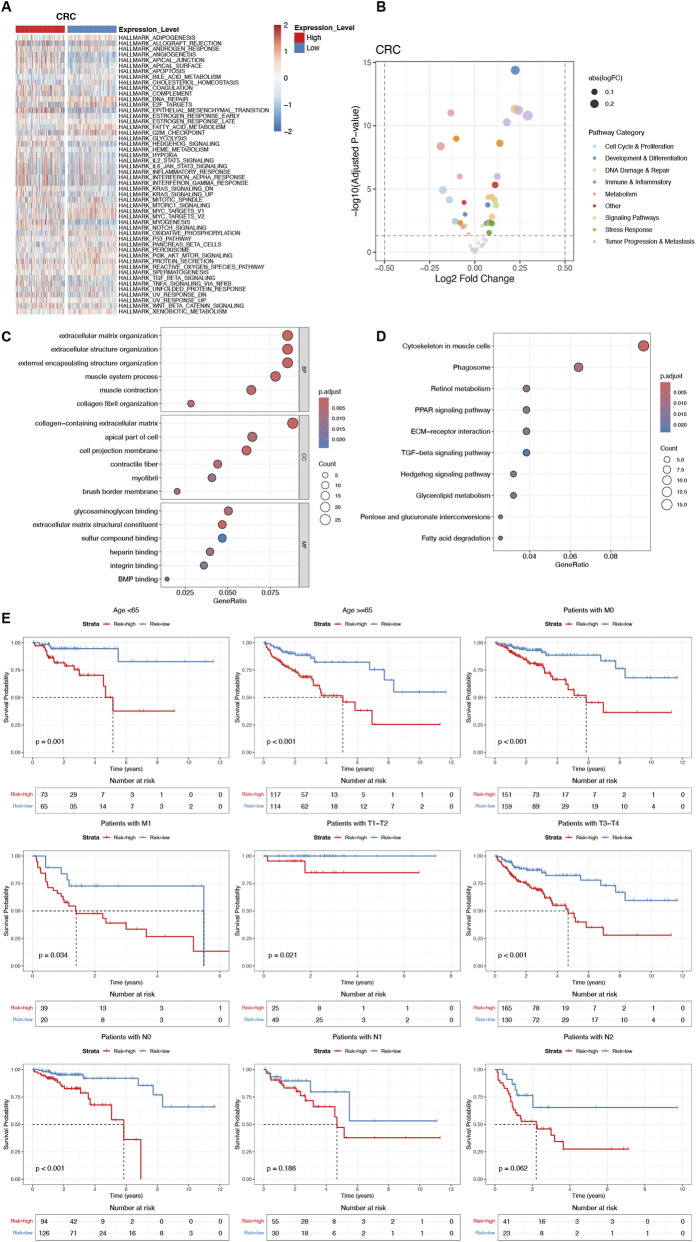
Functional annotation and subgroup prognostic validation of the high-risk subtype in colorectal cancer. **(A)** Heatmap of differential Hallmark pathway activity between the high-risk and low-risk groups. **(B)** Bubble plot of significantly altered pathways between the two groups, classified into major biological domains. Bubble size indicates absolute log2 fold change, and the y-axis indicates statistical significance. **(C)** GO enrichment analysis of differentially expressed genes between the high-risk and low-risk groups. **(D)** KEGG pathway enrichment analysis of differentially expressed genes associated with the high-risk subtype. **(E)** Kaplan-Meier survival analyses across clinicopathological subgroups stratified by age, M stage, T stage, and N stage.

An in-depth examination of the MRS-high subgroup suggested that its unfavorable clinical outcome is closely associated with extensively dysregulated biological alterations. Hallmark pathway analysis revealed a clear distinction from the MRS-low phenotype, with divergent changes involving cell-cycle control, proliferative activity, developmental and differentiation programs, DNA damage and repair machinery, metabolic regulation, stress adaptation, intracellular signaling, and metastatic progression (Figures 3A,B). The results indicate that the high-risk phenotype is not driven by a single oncogenic axis but reflects more comprehensive malignant reconfiguration. Additional functional enrichment analyses reinforced these observations. In the GO analysis, the most significantly enriched terms were mainly related to extracellular matrix organization, structural remodeling of the extracellular compartment, collagen fibrillogenesis, and integrin-related functions (Figure 3C). KEGG annotations showed a similar profile, emphasizing enrichment in ECM-receptor interaction, TGF-β signaling, Hedgehog signaling, PPAR signaling, phagosome-related pathways, and several metabolism-relevant circuits (Figure 3D). Together, the MRS-high subtype is characterized by matrix remodeling, active stromal engagement, and invasion-associated transcriptional programs, all of which plausibly contribute to its poorer prognosis. We next investigated whether MRS holds the consistency of prognostic value across different clinicopathological subgroups. In most strata, including age, M stage, T stage, and N0 disease, Kaplan-Meier analyses showed consistently inferior overall survival among patients assigned to the MRS-high group (Figure 3E). Although the statistical evidence was weakened in the N1 subgroup and remained only borderline in N2, the direction of association remained unchanged. The consistency observed across heterogeneous clinical subsets suggests the MRS as a robust prognostic indicator in a range of clinicopathological settings.

### The MRS-high subtype exhibits a distinct immune-remodeled and stromal-enriched tumor microenvironment

**FIGURE 4:**
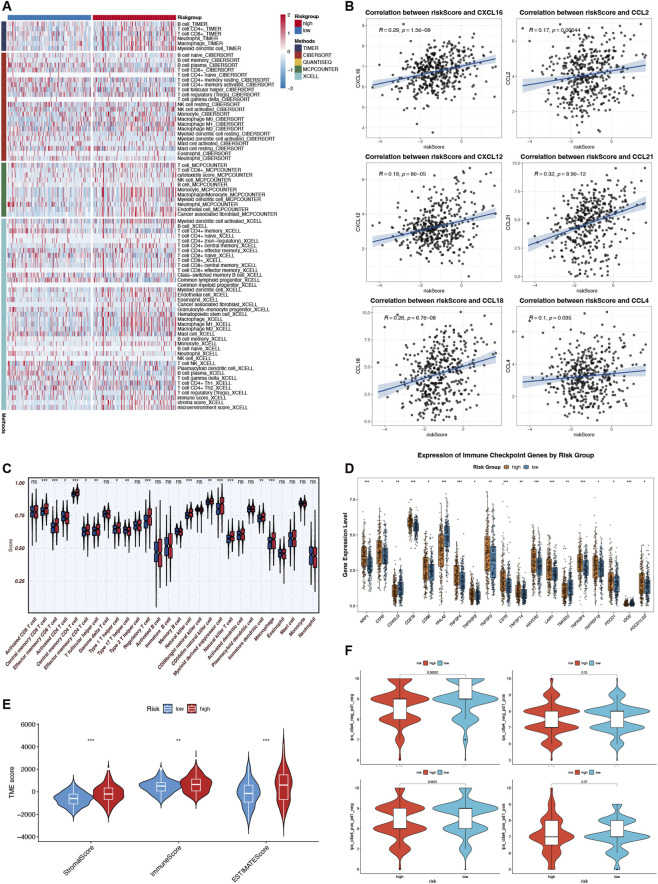
Immune and stromal microenvironmental features associated with the risk signature in colorectal cancer. **(A)** Heatmap showing immune and stromal cell infiltration patterns inferred using multiple deconvolution algorithms, including TIMER, CIBERSORT, quanTIseq, MCP-counter, and xCell. The color gradient represents normalized or scaled relative infiltration estimates within each method and should not be interpreted as directly comparable absolute cell counts across different algorithms. **(B)** Correlation analysis between the MRS score and representative chemokine genes. Correlations were evaluated using Spearman’s rank correlation analysis. The color gradient indicates the strength and direction of correlation, and statistical significance is shown in the corresponding panels. **(C)** Comparison of immune infiltration and immune signature scores between the MRS-high and MRS-low groups. Values represent algorithm-derived relative abundance estimates or normalized immune scores, depending on the corresponding method. **(D)** Expression levels of representative immune checkpoint-associated genes in the MRS-high and MRS-low groups. Values represent normalized transcriptomic expression levels. **(E)** StromalScore, ImmuneScore, and ESTIMATEScore calculated using the ESTIMATE algorithm in the MRS-high and MRS-low groups. Higher scores indicate greater inferred stromal, immune, or combined microenvironmental content. **(F)** IPS-related indices under different immune checkpoint blockade scenarios in the MRS-high and MRS-low groups. Values represent IPS-derived immunogenicity scores. Unless otherwise indicated, two-group comparisons were performed using the Wilcoxon rank-sum test, and statistical significance is indicated in the figure.

Because the MRS-high subtype showed strong matrix-remodeling and invasion-related features, we next examined its tumor microenvironment. Application of several immune-deconvolution approaches disclosed substantial shifts in both immune-cell and stromal-cell composition between the MRS-high and MRS-low strata (Figure 4A), suggesting that the unfavorable MRS state is not confined to tumor-cell-autonomous features alone. The MRS score was positively correlated with CXCL16, CCL2, CXCL12, CCL21, CCL18, and CCL4 (Figure 4B), indicating a chemokine-rich microenvironment associated with enhanced stromal and immune-cell remodeling. Immune infiltration and immune signature scores also differed markedly between groups (Figure 4C). Although the MRS-high group displayed greater immune infiltration, it had worse survival, indicating that this state is more consistent with dysfunctional or restrained immunity than with effective antitumor immune activity. Consistent with this interpretation, the MRS-high group showed increased expression of multiple inhibitory or exhaustion-associated checkpoint molecules, most notably CD274, PDCD1, PDCD1LG2, HAVCR2, LAIR1, and NRP1 (Figure 4D), consistent with an immune-infiltrated yet immunorestrained phenotype. StromalScore, ImmuneScore, and ESTIMATEScore were all significantly elevated in the MRS-high group (Figure 4E), and supplementary analysis showed a relative decrease in epithelial cells together with increased proportions of several immune cell populations (Supplementary Figure S1). IPS-related indices also differed between groups (Figure 4F), and predicted drug-response analysis suggested additional therapeutic differences (Supplementary Figure S2). Together, these results indicate that the MRS-high subtype is embedded within a stromal-rich, immune-remodeled, and likely immunorestrained microenvironment.

### Single-cell analysis localizes the adverse MRS program to epithelial cells with enhanced proliferative and metastatic potential

**FIGURE 5:**
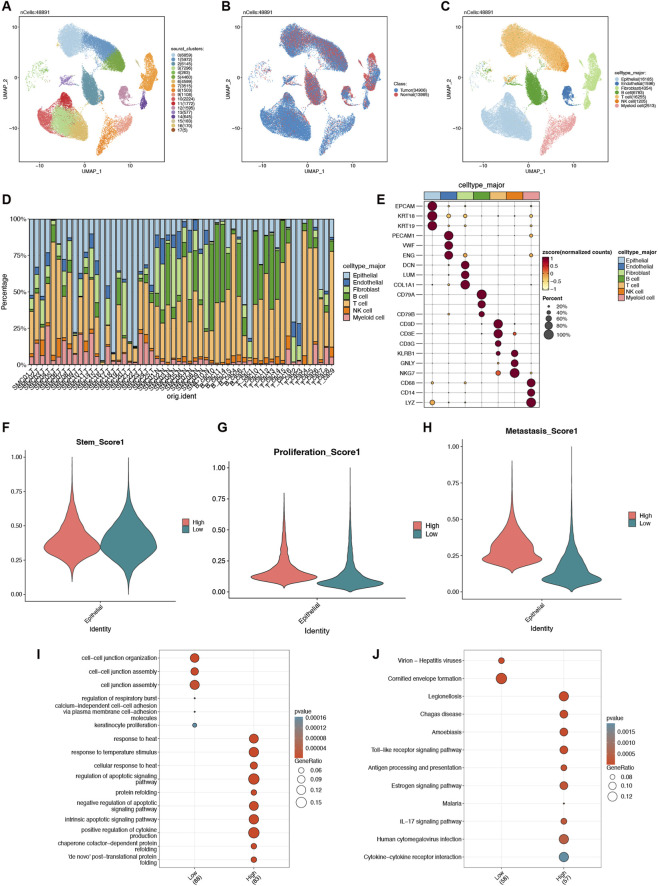
Single-cell dissection of the cellular basis underlying the adverse-risk phenotype in colorectal cancer. **(A)** Uniform manifold approximation and projection (UMAP) visualization of all single cells, showing unsupervised clustering patterns. **(B)** UMAP plot colored by tissue class, distinguishing tumor-derived and normal-derived cells. **(C)** UMAP plot showing major annotated cell lineages in the colorectal cancer microenvironment. **(D)** Stacked bar plot displaying the relative proportions of major cell types across individual samples. **(E)** Dot plot of canonical marker genes used for cell-type annotation. **(F–H)** Violin plots comparing stemness score **(F)** proliferation score **(G)** and metastasis score **(H)** in epithelial cells between the high-state and low-state groups. **(I)** Gene Ontology (GO) enrichment analysis of differentially expressed genes between high-state and low-state epithelial cells. **(J)** Kyoto Encyclopedia of Genes and Genomes (KEGG) pathway enrichment analysis of differentially expressed genes between high-state and low-state epithelial cells.

To define the cellular basis of the adverse MRS phenotype, we analyzed single-cell transcriptomic data from CRC and matched normal tissues. Unsupervised clustering of 48,891 cells identified multiple transcriptionally distinct populations, and tumor-derived and normal-derived cells showed partial segregation in UMAP space (Figures 5A,B). The principal cellular compartments included epithelial cells, endothelial cells, fibroblasts, B lymphocytes, T lymphocytes, NK cells, and myeloid-lineage cells (Figures 5C–E). Additional analyses further showed that, when compared with the MRS-low subset, the MRS-high subset exhibited a lower fraction of epithelial cells but a greater representation of multiple immune populations, notably macrophages/monocytes, CD4 T cells, CD8 T cells, Treg cells, and NK cells (Supplementary Figure S1), consistent with a more profoundly reconstituted tumor microenvironment. We then focused on the epithelial compartment, which most likely represented the malignant population associated with the adverse phenotype. Compared with low-state epithelial cells, high-state epithelial cells showed higher proliferation and metastasis scores, whereas the difference in stemness score was modest (Figures 5F–H). These findings suggest that the MRS-high phenotype is associated with epithelial cells exhibiting enhanced proliferative and metastatic traits, rather than demonstrating that mitoxyperilysis itself directly drives these phenotypes. Functional analysis further supported this interpretation. Low-state epithelial cells were enriched in cell-cell junction and adhesion-related terms, whereas high-state epithelial cells were enriched in stress-response, apoptotic regulation, protein refolding, and cytokine-related processes (Figure 5I). KEGG enrichment analysis indicated that high-state epithelial cells were predominantly associated with Toll-like receptor signaling, antigen processing and presentation, IL-17 signaling, and cytokine-cytokine receptor interaction pathways (Figure 5J). Thus, high-state epithelial cells represent a stress-adaptive and immune-interactive epithelial state associated with the adverse MRS phenotype.

### High-state epithelial programs are associated with globally enhanced cell-cell communication and rewired microenvironmental signaling

**FIGURE 6:**
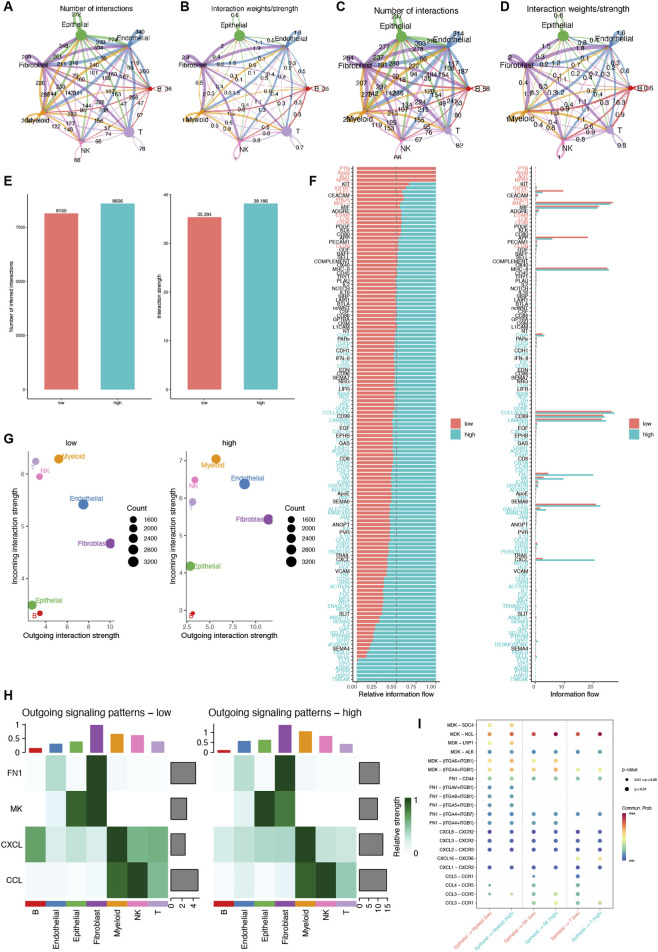
Cell-cell communication analysis reveals enhanced intercellular signaling in the high-state colorectal cancer ecosystem. **(A,B)** Circular plots showing the number **(A)** and strength **(B)** of inferred intercellular interactions in the low-state group. **(C,D)** Circular plots showing the number **(C)** and strength **(D)** of inferred intercellular interactions in the high-state group. **(E)** Comparison of the total number and overall strength of inferred interactions between the two groups. **(F)** Global comparison of signaling pathway information flow between the low-state and high-state groups. **(G)** Outgoing versus incoming interaction strength for each major cell type in both groups. Dot size indicates the total interaction count. **(H)** Heatmaps of representative outgoing signaling patterns, including FN1, MK, CXCL, and CCL. **(I)** Representative ligand-receptor interactions within the MDK, FN1, CXCL, and CCL signaling axes.

After localizing the adverse MRS phenotype to the epithelial compartment, we further examined intercellular communication patterns. Relative to the low-state group, the high-state group displayed a more complex communication network, characterized by both a greater number of inferred interactions and stronger overall interaction intensity (Figures 6A–E). The high-state communication landscape was quantitatively more expansive, exhibiting both a larger number of inferred interactions (8,606 vs. 8,150) and a stronger aggregate interaction intensity (38.186 vs. 35.394), a pattern consistent with globally heightened intercellular signaling. Stromal and immune compartments constituted major signaling hubs within the tumor microenvironment, whereas epithelial cells displayed a more prominent interactive role in the high-state setting (Figure 6G). These findings argue against the assumption that MRS-high epithelial cells simply act as recipients to extrinsic microenvironmental cues. Rather, they appear to actively participate in remodeling the surrounding tumor ecosystem. Their increased signaling activity may further contribute to the reinforcement of pro-tumor circuitry and establish more intensive epithelial-stromal-immune crosstalk. Rewiring of communication was also evident at the pathway level, where FN1-, MK-, CXCL-, and CCL-associated signaling displayed pronounced redistribution (Figures 6F,H). This remodeling was further identified in multiple ligand-receptor pairs, including MDK-SDC4, MDK-NCL, MDK-LRP1, FN1-CD44, FN1-integrin complexes, CXCL family-CXCR2, and CCL family-CCR1/CCR5 interactions (Figure 6I). Overall, the MRS-high phenotype represents not only a state of intrinsic epithelial aggressiveness, but also altered intercellular communication landscape that links epithelial activity with stromal reprogramming and inflammatory niche formation.

### ANO1 emerges as a downstream effector associated with the MRS-high phenotype in colorectal cancer

**FIGURE 7:**
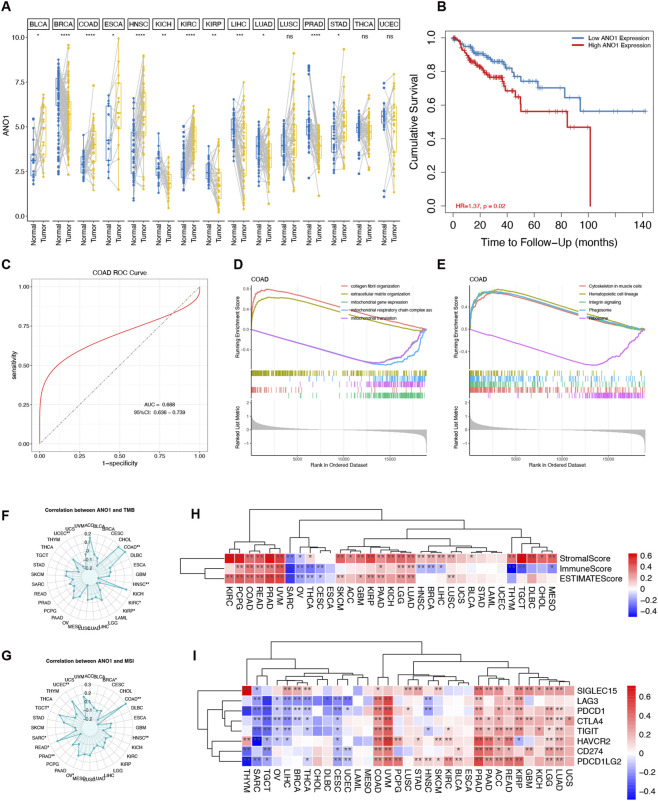
Clinical, transcriptomic, and immunogenomic landscape of ANO1 in colorectal cancer and across cancers. **(A)** Pan-cancer comparison of ANO1 expression between tumor and normal tissues. **(B)** Kaplan-Meier survival analysis of patients with high and low ANO1 expression in COAD. **(C)** ROC curve evaluating the diagnostic value of ANO1 in distinguishing COAD from normal tissues. **(D,E)** Representative GSEA plots showing pathways associated with ANO1 expression in COAD. **(F)** Correlation between ANO1 expression and tumor mutational burden (TMB) across cancers. **(G)** Correlation between ANO1 expression and microsatellite instability (MSI) across cancers. **(H)** Correlations between ANO1 expression and tumor microenvironment-related scores across cancers. **(I)** Correlations between ANO1 expression and representative immune checkpoint-related genes across cancers.

To identify a candidate downstream effector associated with the adverse epithelial state, we focused on ANO1. Pan-cancer analysis showed that ANO1 was aberrantly expressed across multiple tumor types, including COAD, compared with normal tissues (Figure 7A). In COAD, elevated ANO1 expression was significantly associated with poorer survival outcomes (HR = 1.37, p = 0.02; Figure 7B), and ROC analysis showed moderate diagnostic value for distinguishing tumor from normal tissues (AUC = 0.688, 95% CI, 0.636–0.739; Figure 7C). GO analysis showed that ANO1-associated transcriptional states were enriched in collagen fibril organization, extracellular matrix organization, and related structural processes (Figure 7D). KEGG analysis further highlighted integrin signaling, phagosome, hematopoietic lineage-related programs, and cytoskeleton-associated pathways (Figure 7E). These results suggest that ANO1 is linked to matrix remodeling, immune-associated signaling, and structural adaptation, in line with the broader MRS-high phenotype. ANO1 was also associated with immunogenomic and microenvironment-related features. Pan-cancer radar analyses showed correlations with TMB and MSI, including significant associations in COAD (Figures 7F,G). Furthermore, ANO1 expression showed broad correlations with StromalScore, ImmuneScore, and ESTIMATEScore across multiple cancer types (Figure 7H). ANO1 was likewise positively associated with several representative inhibitory immune checkpoint molecules, including PDCD1, CTLA4, TIGIT, HAVCR2, and CD274 (Figure 7I). These findings suggest that ANO1-high tumors may reside in an immune-infiltrated but functionally restrained microenvironment, consistent with the poorer predicted immunotherapy response observed in the MRS-high subgroup.

### ANO1 depletion suppresses colorectal cancer growth and invasiveness both *in vitro* and *in vivo*

**FIGURE 8:**
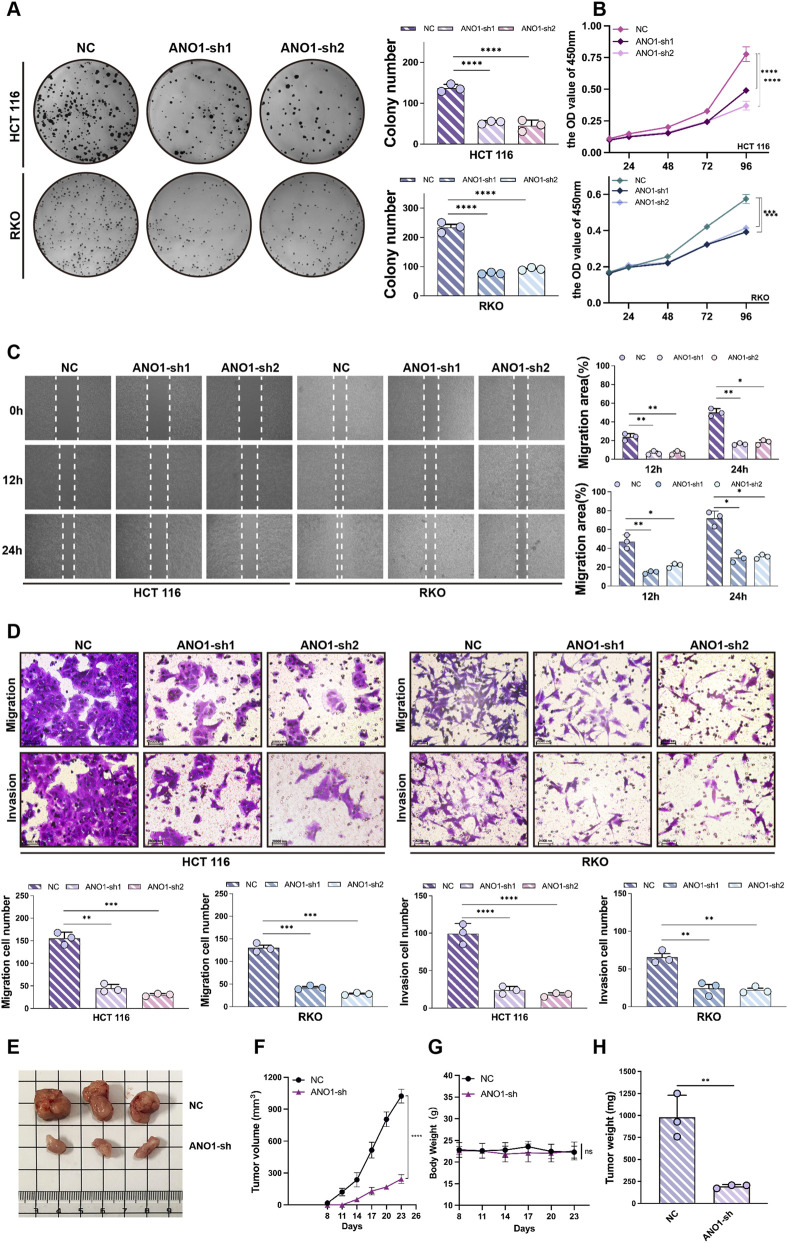
Functional validation demonstrates that ANO1 promotes malignant phenotypes of colorectal cancer cells *in vitro* and *in vivo*. **(A)** Representative colony formation images and quantification in HCT116 and RKO cells transduced with negative control (NC) or two independent ANO1-targeting shRNAs (ANO1-sh1 and ANO1-sh2). **(B)** CCK-8 assays showing cell proliferation of HCT116 and RKO cells following ANO1 knockdown. Optical density (OD) values at 450 nm were measured at the indicated time points. **(C)** Representative wound-healing images at 0, 12, and 24 h, together with quantification of migration area, in HCT116 and RKO cells with or without ANO1 knockdown. **(D)** Representative Transwell migration and invasion images and corresponding quantification in HCT116 and RKO cells transduced with NC, ANO1-sh1, or ANO1-sh2. **(E)** Representative images of xenograft tumors formed by CRC cells expressing NC or ANO1-targeting shRNA. **(F)** Growth curves of xenograft tumors in the NC and ANO1-knockdown groups. **(G)** Body weight curves of mice during the xenograft experiment. **(H)** Final tumor weights in the NC and ANO1-knockdown groups. Data are presented as mean ± SD. For *in vitro* comparisons among NC, ANO1-sh1, and ANO1-sh2 groups, one-way ANOVA followed by Tukey’s multiple-comparison test was used unless otherwise indicated. Tumor growth curves were analyzed using repeated-measures analysis, and final tumor weight was compared using an unpaired two-tailed Student’s t-test. Statistical significance is indicated in the figure.

Western blot analysis confirmed that ANO1 protein expression was markedly reduced by both ANO1-sh1 and ANO1-sh2 in HCT116 and RKO cells, with GAPDH used as the loading control (Supplementary Figure S3A). Using these validated ANO1-deficient cell models, we found that ANO1 knockdown significantly impaired clonogenic growth and proliferative capacity, as shown by colony formation and CCK-8 assays (Figures 8A,B). ANO1 depletion also delayed wound closure and reduced the migratory and invasive abilities of CRC cells in Transwell assays (Figures 8C,D). Consistently, in the xenograft model, tumors derived from ANO1-depleted cells grew more slowly and showed reduced final tumor weight, while mouse body weight remained comparable between groups (Figures 8E–H). To further support the clinical relevance of ANO1, we performed immunohistochemical analysis in an institutional CRC cohort. Representative images showed stronger ANO1 staining in tumor tissues than in adjacent non-tumor tissues (Supplementary Figure S3B). Among 68 cases with complete clinicopathological information and evaluable ANO1 staining, higher ANO1 expression was associated with nodal involvement, distant metastasis, and advanced clinical stage, whereas its association with T stage was less pronounced (Supplementary Figures S3C–F). Collectively, these functional and tissue-level validation results support ANO1 as an important downstream effector associated with malignant CRC phenotypes and the adverse MRS-related state.

## Discussion

In this study, we developed a mitoxyperilysis-related risk signature (MRS) that reproducibly stratified survival in CRC across independent cohorts. The MRS also showed translational potential by supporting construction of a clinically interpretable nomogram for individualized prognosis. More importantly, the MRS captured a coherent biological state rather than a purely statistical classification. The MRS-high subtype was characterized by ECM remodeling, stromal activation, immune reconfiguration, epithelial-state heterogeneity, and intensified cell-cell communication.

The most important conceptual advance of this study is that mitoxyperilysis-related transcriptional programs emerge as a clinically interpretable axis in CRC. In the original Cell report, mitoxyperilysis was characterized as a lytic stress response triggered by the convergence of innate immune activation and metabolic disruption (Wang et al., 2025). While our analyses do not constitute direct evidence that mitoxyperilysis occurs uniformly across CRC lesions, they do indicate that transcriptional states aligned with this pathway identify a biologically integrated tumor condition marked by oxidative stress, inflammatory signaling, and metabolic derangement (Chen et al., 2024; Saxena et al., 2024). Viewed in this way, the MRS shifts mitoxyperilysis from the level of a mechanistic cell death phenomenon to that of a systems-level framework for understanding aggressive tumor states in CRC (Saxena et al., 2024; Wang et al., 2025).

Rather than being a collection of isolated malignant features, the MRS-high subtype reflects coordinated tumor state. In this subtype, matrix remodeling, invasive behavior, and immune dysfunction do not occur in isolation but instead arise within a shared biological context. Across enrichment analyses, extracellular structural reorganization, collagen-related processes, and integrin-mediated signaling consistently stood out as the dominant features, situating this subtype in a microenvironment that is both stromally supportive and conducive to invasion. Taken together, these findings suggest clear clinical implications. Extracellular matrix remodeling is increasingly recognized as an active driver of tumor behavior, with significant effects on enhancing mechanotransductive signaling, fostering metastatic capacity, and diminishing therapeutic susceptibility (Prakash and Shaked, 2024). Against this background, the unfavorable survival observed in the MRS-high group becomes biologically coherent, as tumors embedded in sustained stromal activation and ongoing matrix reorganization would be expected to acquire the traits that drive local invasion, dissemination, and progressive disease evolution (An et al., 2024; Guan et al., 2026).

Importantly, the stromal remodeling observed in MRS-high subtype may also help explain an apparent paradox, where these tumors appear more immune-infiltrated yet remain functionally constrained. In our study, MRS-high tumors showed more extensive immune infiltration and higher ImmuneScore and ESTIMATEScore, yet were associated with worse prognosis and reduced predicted responsiveness to immunotherapy. While this pattern fails to fit a simple immune-desert phenotype and instead reflects an immune-infiltrated but ineffective state, it may arise from a matrix-rich and stromally activated microenvironment that allows immune-cell recruitment but restricts productive antitumor activity through checkpoint upregulation, suppressive myeloid polarization, and stromal exclusion simultaneously (Chuang et al., 2024). This interpretation is aligned with the current understanding of CRC immunobiology, particularly in MSS disease, where the presence of immune cells alone does not necessarily guarantee the translation of effective antitumor immunity (Guven et al., 2024). The positive association between the MRS-high state and checkpoint-related programs fits well within this framework, pointing to immune restraint rather than immune competence (Qu et al., 2024), and is further supported by growing evidence that suppressive myeloid states and stromal barriers are key roles in limiting anti-tumor immunity in CRC (Zeng et al., 2024). Collectively, the MRS-high subtype represents a stromalized and immunorestrained malignant state, in which matrix remodeling, metastatic potential, and ineffective immune engagement reinforce one another (Wang et al., 2024).

Our single-cell analyses suggest that the adverse MRS phenotype is not broadly distributed across the tumor ecosystem, but is primarily confined to a distinct epithelial population. Specifically, these MRS-high epithelial cells showed increased proliferative and metastatic potential, along with stress-adaptive, cytokine-related, and immune-interactive programs. Rather than representing a rapidly growing malignant population, they appear to be adapted to a hostile metabolic and inflammatory niche while also obtaining the capacity to actively reshape their surroundings. This is in line with mitoxyperilysis-associated stress biology, where mitochondrial and metabolic stress alters the susceptibility to cell death and promotes epithelial populations with greater survival capacity, inflammatory signaling output, and microenvironmental plasticity (Wang et al., 2025). Within this biological setting, the MRS- high epithelial compartment may represent the key population in which mitoxyperilysis-related stress underlies aggressive tumor behavior. These epithelial cells did not act in isolation. Cell-cell communication analysis showed that they were actively involved in the signaling networks of the tumor ecosystem, particularly through FN1-, MK-, CXCL-, and CCL-associated pathways (Jin et al., 2021). The result further indicates that MRS-high epithelial cells may help shape a permissive microenvironment, where malignant epithelial aggressiveness is accompanied by fibroblast activation, endothelial support, immune-cell recruitment, and inflammatory remodeling (Ma et al., 2025). Overall, these findings suggest that stress-adapted malignant epithelial cells are intrinsically aggressive and capable of creating a microenvironment that favors tumor progression. Therefore, the concurrent presence of invasive behavior, stromal activation, immune infiltration, and functional immune restraint in MRS-high phenotype likely reflects a shared underlying biological basis rather than a set of separate features (Peng and Zhan, 2025; Su et al., 2025).

Within the MRS-defined state, ANO1 should be interpreted with caution. Our data support ANO1 as a downstream effector associated with the MRS-high state, with both clinical and biological significance, and little evidence that it acts as the initiating driver. High ANO1 expression was associated with poor prognosis, together with matrix-remodeling, immunogenomic heterogeneity, and activation of inhibitory immune checkpoint programs. Functionally, ANO1 depletion suppressed clonogenicity, proliferation, migration, invasion, and xenograft growth. These findings are consistent with emerging evidence that ANO1 contributes to tumor progression and therapy resistance in gastrointestinal malignancies (Jiang et al., 2023). They are also in line with recent reviews emphasizing the central role of ANO1 in gastrointestinal cancer biology (Zhang et al., 2025). At the same time, our results indicate that ANO1 should be understood within the broader MRS-defined biological state rather than as a substitute for it (Hu Y. et al., 2025; Terzi et al., 2025).

This study has several limitations. Because the MRS was developed from retrospective transcriptomic datasets, our findings primarily support associations between mitoxyperilysis-related transcriptional programs and aggressive, immune-remodeled CRC states, rather than direct causality. Further prospective, spatially resolved, and mechanistic studies are needed to determine whether mitoxyperilysis itself contributes causally to stromal remodeling, immune restraint, and tumor progression (George and Johdi, 2025).

In summary, our study shows that mitoxyperilysis-related transcriptional programs define a clinically aggressive and immune-remodeled subtype of CRC. The MRS shows great prognostic value and highlights matrix and microenvironmental remodeling. Moreover, it identifies a high-state epithelial population as the pivotal compartment at the single-cell resolution and points to its role in active intercellular communication. Within this state, ANO1 functions as a downstream effector closely relevant to malignant progression. More broadly, these results provide that mitochondrial stress-associated biology may shape CRC progression and support the mitoxyperilysis axis as a promising conceptual framework for future prognostic modeling and therapeutic investigation.

## Data Availability

The original contributions presented in the study are included in the article/Supplementary Material, further inquiries can be directed to the corresponding authors.
