# Supplementary material for: A mitoxyperilysis-related signature stratifies prognosis and identifies an aggressive colorectal cancer ecosystem with immune remodeling
Source: Front Cell Dev Biol. 2026 Jul 3;14:1851988. doi: 10.3389/fcell.2026.1851988 (PMC13376262; doi:10.3389/fcell.2026.1851988)
Supplement: Supplementary file 1 [file DataSheet1.docx]

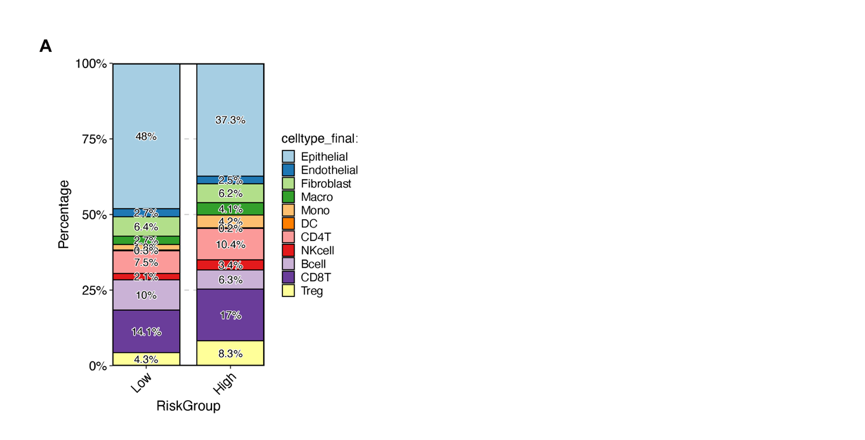
Figure S1. Differences in major cell-type composition between the low-risk and high-risk groups.

(A) Stacked bar plot showing the relative proportions of major cell populations in the low-risk and high-risk groups.


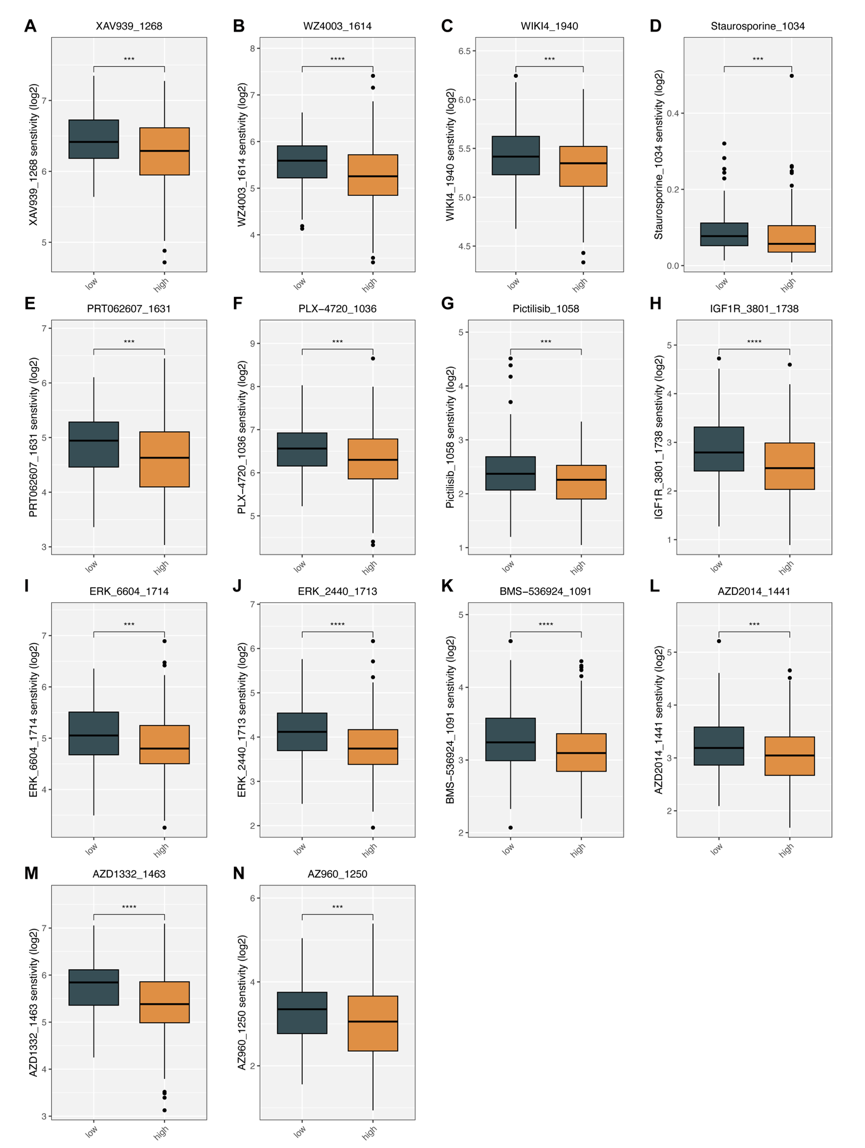
Figure S2. Comparison of predicted drug response profiles between the low-risk and high-risk groups.


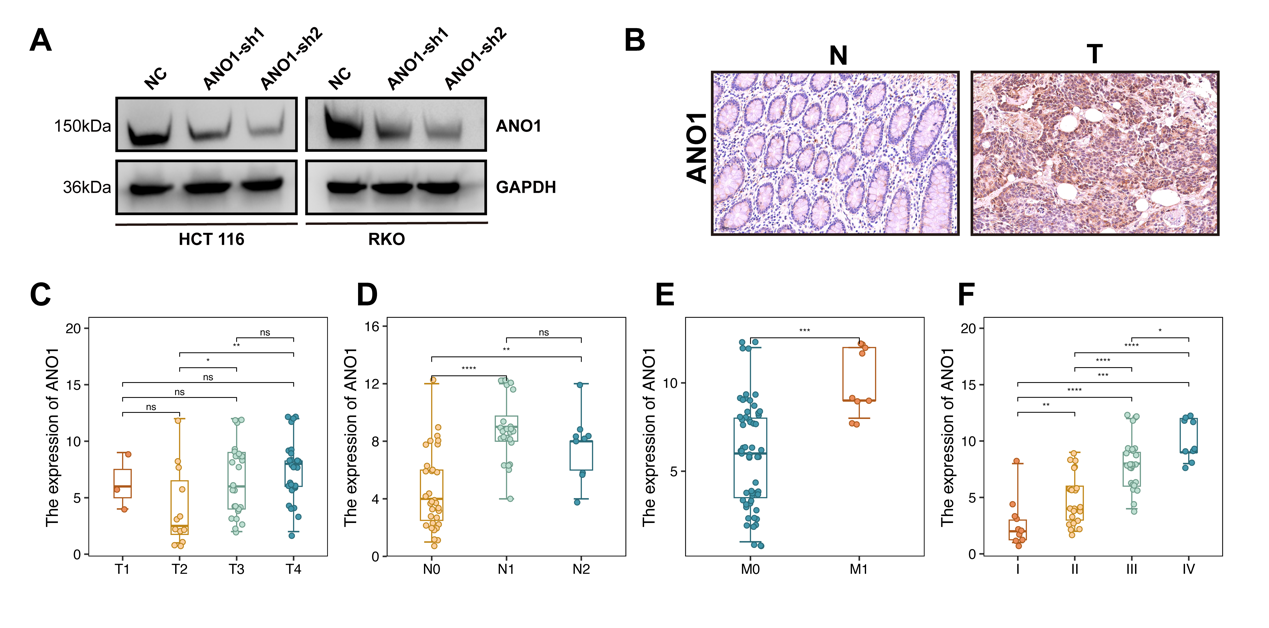
Figure S3. Supplementary validation of ANO1 knockdown efficiency and tissue-level ANO1 expression in colorectal cancer.

(A) Western blot analysis confirming ANO1 knockdown efficiency in HCT116 and RKO cells transduced with negative-control shRNA (NC), ANO1-sh1, or ANO1-sh2. GAPDH was used as the loading control. (B) Representative immunohistochemical staining images showing ANO1 expression in adjacent non-tumor colorectal tissue (N) and colorectal cancer tissue (T). (C-F) Association between ANO1 expression and clinicopathological parameters in the institutional CRC cohort with complete clinical information and evaluable ANO1 IHC staining. ANO1 expression levels were compared according to T stage (C), N stage (D), M stage (E), and overall clinical stage (F). Each dot represents an individual case. Box plots show the median and interquartile range, with whiskers indicating the data range. For two-group comparisons, the Wilcoxon rank-sum test was used; for comparisons among more than two groups, the Kruskal-Wallis test followed by pairwise comparisons was applied. ns, not significant; *P < 0.05; **P < 0.01; ***P < 0.001; ****P < 0.0001.
